# Hypoattenuating Leaflet Thickening After Transcatheter Pulmonary Valve Implantation: An Urgent Call to Action

**DOI:** 10.1016/j.jaccas.2025.103889

**Published:** 2025-07-03

**Authors:** Pietro Spagnolo, Francesca Bevilacqua, Noemi Perillo, Angelo F. d’Aiello, Giulia Guglielmi, Giulia Pasqualin, Angelo Micheletti, Diana G. Negura, Massimo Chessa

**Affiliations:** a Unit of Radiology, IRCCS Policlinico San Donato, San Donato Milanese, Milan, Italy; b Adult Congenital Heart Disease (ACHD) Unit, IRCCS Policlinico San Donato, San Donato Milanese, Milan, Italy; c Vita-Salute San Raffaele University, Milan, Italy

**Keywords:** computed tomography, hypoattenuated leaflet thickening, new-generation device, self-expanding pulmonary valve, transcatheter pulmonary valve implantation

## Abstract

The Venus P-Valve is a novel self-expanding prosthesis designed for transcatheter pulmonary valve implantation (TPVI) in patients with severe pulmonary regurgitation and dilated right ventricular outflow tracts. This case series assessed the incidence of subclinical leaflet thrombosis, defined as hypoattenuated leaflet thickening (HALT), and reduced leaflet motion 6 months after TPVI in 7 patients treated between January and April 2023. HALT was detected via computed tomography angiography in 71% of patients, primarily affecting the right cusp. Despite the high incidence of HALT, no significant valve dysfunctions or adverse clinical events were documented. Patients with HALT were treated with oral anticoagulation, resulting in a reduction of HALT at follow-up. These findings underscore the uncertainty regarding the short-term clinical relevance of HALT and reduced leaflet motion, emphasizing the need for individualized antithrombotic strategies. Larger studies are required to evaluate the long-term impact and optimal therapeutic approach for patients undergoing TPVI with the Venus P-Valve.

## Graphical abstract

**Figure:**
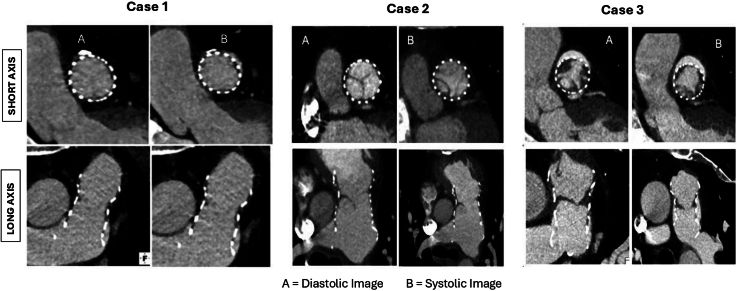

The Venus P-Valve (Medtech) is a self-expanding nitinol frame partially covered with porcine pericardium designed for patients with severe pulmonary regurgitation (PR) and dilated native right ventricular outflow tracts (RVOTs).^1^ Despite promising early results, the optimal post-implantation antithrombotic therapy remains unclear. Subclinical leaflet thrombosis, detected as hypoattenuated leaflet thickening (HALT) on computed tomography angiography (CTA), may cause reduced leaflet motion (RELM), increased transvalvular gradients, and adverse outcomes after both surgical and transcatheter bioprosthetic aortic valves procedures.^2^

**Take-Home Messages**

• Hypoattenuating leaflet thickening after transcatheter pulmonary valve implantation with the Venus P-Valve represents an emerging issue that needs to be identified, adequately managed, and, if possible, prevented.
• To ensure optimal post-transcatheter pulmonary valve implantation outcomes, patients should undergo comprehensive imaging, including computed tomography angiography, to assess valve position, function, and to detect hypoattenuating leaflet thickening.
• A tailored antithrombotic regimen is essential to minimize thromboembolic risk and maintain valve functionality.

This case series evaluated HALT and RELM incidence in 7 patients undergoing transcatheter pulmonary valve implantation (TPVI) with the Venus P-Valve between January and April 2023. It was approved by the institutional ethics committee, and written informed consent was obtained from all patients. Preprocedural CTA and cardiac magnetic resonance imaging were used to assess pulmonary dimensions and ventricular volumes (Table 1).

**Table 1:** Demographic and Procedural Data

	Patient No.						
	1	2	3	4	5	6	7
Sex	M	F	M	M	F	F	M
CHD diagnosis	TOF	Valvular and subvalvular PS	TOF	TOF	TOF	Fallot-type DORV	TOF
Palliative treatment	Left mBTS	No	No	No	Left mBTS	No	No
Surgery	TAP	TAP + PVC	TAP	TAP	TAP	TAP	Separate patches on the infundibulum and MPA + PVC
Age at TPVI, y	58	41	33	38	33	44	60
BMI at TPVI, kg/m^2^	18.7	25.0	21.2	23.5	19.2	24.1	25.3
NYHA functional class	III	II-III	II	II	II-III	II	II
CMR RV EDVi, mL/m^2^	160	92	140	145	123	162	116
CMR RV ESVi, mL/m^2^	87	42	82	48	78	101	48
CMR RV EF, %	46	55	41	48	37	35	59
CMR RV EDVi/LV EDVi	2.17	1.3	1.75	1.54	2.82	1.59	1.2
CMR PRF, %	51	45	53	50	52	44	59
CTA RVOT d, mm AP × LL	41 × 39	40 × 34	38 × 28	42 × 29	38 × 32	47 × 31	34 × 28
CTA PV anulus d, mm, SI × LL	17 × 40	24 × 27	34 × 42	35 × 39	27 × 37	30 × 32	34 × 28
CTA mid-part MPA d, mm SI/AP × LL	44 × 40	29 × 34	31 × 39	38 × 35	33 × 34	25 × 33	32 × 32
CTA prebifurcation part MPA d, mm SI × LL	42 × 47	34 × 50	35 × 38	36 × 40	45 × 36	20 × 24	29 × 40
CTA MPA length, mm	64	27	37	43	20	48	40
CTA LPA d, mm, SI × LL	28 × 32	20 × 20	30 × 31	27 × 26	12 × 13	13 × 16	30 × 38
CTA RPA d (mm, SI x LL)	34 × 37	23 × 21	29 × 34	26 × 30	37 × 40	21 × 24	22 × 23
Venus P-Valve size, mm	32-25	32-25	32-25	36-25	30-25	32-25	30-25
Antithrombotic therapy after TPVI	SAPT aspirin	SAPT clopidogrel	SAPT aspirin	SAPT aspirin	SAPT aspirin	OAC Eliquis 5 mg twice a day	SAPT aspirin

AP = antero-posterior; BMI = body mass index; CHD = congenital heart disease; CMR = cardiac magnetic resonance; CTA= computed tomography angiography; d = dimensions; DORV = double outlet right ventricle; EDVi = end-diastolic volume indexed; ESVi = end-systolic volume indexed; EF = ejection fraction; F = female; LL = latero-lateral; LPA = left pulmonary artery; LV = left ventricle; M = male; mBTS = modified Blalock-Taussig shunt; MPA = main pulmonary artery; OAC = oral anticoagulant therapy; PRF = pulmonary regurgitant fraction; PS = pulmonary stenosis; PV = pulmonary valve; PVC = pulmonary valve commissurotomy; RPA = right pulmonary artery; RV = right ventricle; SAPT = single anti-platelet therapy; SI = superior-inferior; TAP = transannular patch; TOF = tetralogy of Fallot; TPVI = transcatheter pulmonary valve implantation.

The implantation was performed under general anesthesia with fluoroscopic guidance using heparin to maintain an activated clotting time of 200 to 220 seconds. Balloon interrogation of the RVOT with a 34-mm compliant Amplatzer Sizing Balloon catheter determined the valve size, which exceeded the balloon’s waist by 2 to 4 mm, with the mid-body length matched to the main pulmonary artery length. All procedures were successfully completed without immediate complications.

Postoperatively, 5 patients received aspirin (100 mg/d), 1 received clopidogrel (75 mg/d) due to aspirin intolerance, and 1 continued oral anticoagulation (Eliquis 5 mg twice daily).

As part of post-market surveillance protocol, CTA and transthoracic echocardiogram were performed at 6 months. CTA was performed using an electrocardiogram–gated Siemens Dual Source 256-slice scanner with multiplanar reconstructions for a comprehensive assessment of valvular morphology and function. HALT was defined as hypoattenuated leaflet thickening, with or without rigidity, detected in at least 2 different projections (long- and short-axis) and 2 reconstruction time intervals (systole and diastole) (Figure 1).^3^ The affected cusps were classified as anterior (A), left (L), or right (R), and their number and maximum thickness at mid-diastole were measured.

**Figure 1:**
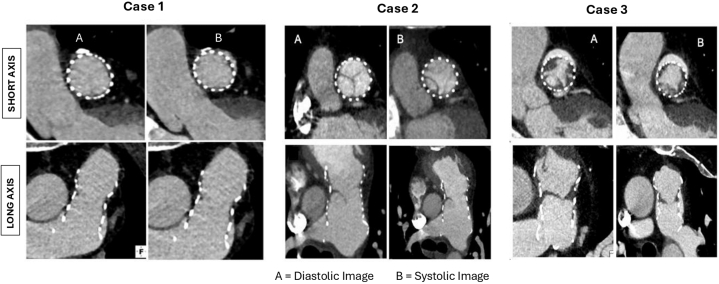
2-Dimensional Cardiac Computed Tomography Images Two-dimensional images in short-axis and long-axis in diastole (A) and systole (B). Case 1 shows an example without hypoattenuating leaflet thickening (HALT). Case 2 shows mild HALT on a single valve leaflet motion. Case 3 shows significant HALT on all 3 leaflets with concomitant restricted leaflets motion.

CTA detected HALT in 5 patients (71%), with all 3 leaflets affected in 3 cases. Leaflet rigidity was observed in 71%, predominantly in the right cusp, with a mean leaflet thickness of 4.0 ± 1.7 mm (range, 1 to 6 mm). Of the 2 patients without HALT, 1 was on anticoagulant therapy.

Echocardiography confirmed proper valve positioning and function, with a mean transvalvular gradient of 9.4 ± 3 mm Hg and no significant PR at follow-up. No patients experienced heart failure symptoms or thromboembolism on CTA. Patients diagnosed with HALT received oral anticoagulation therapy, which resulted in a progressive resolution of HALT findings on follow-up CTA at 12 months post-procedure.

## Patient characteristics and procedural outcomes

Patient 1 was a 58-year-old man with tetralogy of Fallot (TOF) diagnosed at birth who underwent a left modified Blalock-Taussig shunt at 9 months and ventricular septal defect (VSD) closure with a Dacron patch and RVOT reconstruction with a pericardial transannular patch (TAP) at 2 years. His history was complicated by ventricular arrhythmias, heart failure symptoms, and declining functional capacity. Echocardiography showed mildly reduced biventricular function, right ventricular (RV) dilatation, and severe PR without stenosis. A multidisciplinary team recommended TPVI to improve his condition.

He received a Venus P-Valve 30/25 mm and was discharged on single antiplatelet therapy (SAPT) (aspirin 100 mg daily) (Table 1). At the 6-month follow-up, CTA showed proper valve position, HALT in all 3 leaflets, and RELM in 2 (A and R, Video 1). Echocardiography revealed a mildly reduced RV ejection fraction (45%), peak/mean gradients of 30/15 mm Hg across the valve, and no pulmonary regurgitation (Table 2).

**Table 2:** Follow-up Data 6 Months After TPVI

	Patient No.						
	1	2	3	4	5	6	7
CTA (days after-TPVI)	193	220	175	173	189	197	178
HALT leaflet A	x	—	x	x	—	—	x
HALT leaflet R	x	x	x	x	—	—	x
HALT leaflet L	x	x	x	—	—	—	x
RELM leaflet A	x	—	—	—	—	—	—
RELM leaflet R	x	—	x	x	—	—	x
RELM leaflet L	—	x	x	—	—	—	—
Maximal leaflet thickness, mm							
A	6	1	3	1	0.5	0,5	4
R	6	2	3	6	0.6	0,5	4
L	4	3	5	2	1	0,5	4
Maximum/medium pressure gradient (echo), mm Hg	30/15	18/11	18/12	10/5	15/6	15/8	23/9
Residual pulmonary regurgitation (echo)	Mild	Mild	Mild	Mild	Mild	Mild	Mild
Adverse clinical events	No	No	No	No	No	No	No

A + anterior; CTA = computed tomography angiography; echo = echocardiography; HALT = hypoattenuated leaflet thickening; L = left; R = right; RELM = reduced leaflet motion; TPVI = transcatheter pulmonary valve implantation.

Patient 2 was a 41-year-old woman with neonatal valvular and subvalvular pulmonary stenosis who underwent a pulmonary valve commissurotomy at 9 months. She presented with heart failure and severe PR. Post-TPVI with a Venus P-Valve 30/25 mm, she was discharged on clopidogrel. At 6 months, CTA revealed HALT in 2 leaflets (R and L) and RELM in only 1 leaflet (L), with echocardiography showing gradients of 18/11 mm Hg and no PR.

Patient 3 was a 33-year-old man with neonatal TOF who underwent VSD closure and RVOT reconstruction at 10 months. Post-TPVI with a Venus P-Valve 32/25 mm, he was discharged on aspirin. At 6 months, CTA revealed HALT in all 3 leaflets and RELM in 2 (L and R), with echocardiography showing gradients of 18/12 mm Hg and mild PR.

Patient 4 was a 38-year-old man with neonatal TOF who underwent VSD closure and RVOT reconstruction at 12 months, followed by residual VSD closure at 8 years. Post-TPVI with a Venus P-Valve 36/25 mm, he was discharged on aspirin. At 6 months, CTA showed HALT in 2 leaflets (A and R) and RELM in only 1 leaflet (R), with gradients of 10/5 mm Hg and no PR.

Patient 5 was a 33-year-old woman with TOF and left pulmonary artery (LPA) hypoplasia who underwent TAP and LPA patch enlargement at 8 months. Post-TPVI with a Venus P-Valve 30/25 mm and LPA stenting, she was discharged on aspirin. At 6 months, no HALT was observed with gradients of 15/5 mm Hg and no PR.

Patient 6 was a 44-year-old woman with Fallot-type double RVOT who underwent TAP at 18 months. Post-TPVI with a Venus P-Valve 32/25 mm, she was discharged on Eliquis. At 6 months, CTA showed no HALT with gradients of 15/8 mm Hg and mild PR.

Patient 7 was a 60-year-old man with neonatal TOF who underwent RVOT and pulmonary artery enlargement at 23 months. Post-TPVI with a Venus P-Valve 30/25 mm, he was discharged on aspirin. At 6 months, CTA revealed HALT in all 3 leaflets and RELM in 1 (R) with gradients of 23/9 mm Hg and mild PR.

## Discussion

The Venus P-valve is a self-expandable prosthesis demonstrating promising outcomes for patients with significant PR and large RVOTs offering an effective treatment option. Clinical studies have reported favorable outcomes at medium-term follow-ups. Echocardiographic evaluations have consistently shown sustained improvements in hemodynamic parameters. At follow-up intervals of 6 to 12 months, studies have shown a significant reduction in PR and stabilization of the RVOT gradient. RV function showed marked improvement, with normalization of tricuspid annular plane systolic excursion values in most patients.^4^ Patients also reported improved exercise capacity and quality of life following the procedure. However, long-term outcomes remain under investigation because data beyond 5 years are still limited. Some complications have been documented, including a small number of cases of infective endocarditis and infolding of the valve, necessitating careful post-procedural monitoring.^5^ Although transthoracic echocardiography reliably measures transvalvular pressure gradients, it cannot provide detailed prosthetic leaflet assessments, where CTA with 2-dimensional and 3-dimensional imaging offers a more comprehensive evaluation.

HALT has been identified as a significant event in transcatheter and surgical aortic valves.^2^ A recent small series has shown an 83.3% incidence of HALT at 30 days after TPVI with the Sapien3 valve^6^ and Harmony (Medtronic) valves.^7^ Despite dual antiplatelet therapy, a single case of Venus P-Valve leaflet microthrombosis was also documented 6 months post-procedure.^8^

Our case series reported a higher incidence of HALT compared to aortic valve studies, which document rates of up to 40%,^9^ but the findings are consistent with the limited data available in the literature on TPVI.^5^

At the 6-month follow-up, despite the high incidence of HALT and RELM observed in our population, no patient developed significant valve dysfunction in terms of stenosis, regurgitation, or durability. Only 1 case (patient 1) showed a mild increase in transvalvular gradients.

Additional studies are required to determine whether undiagnosed and untreated HALT could contribute to the premature deterioration of TPVI. This is especially significant for patients with congenital heart disease, where the long-term durability of the device plays a crucial role in their overall treatment pathway.

This finding underscores the uncertainty surrounding the short-term (6-month) clinical relevance of HALT, highlighting the need for further investigation in larger studies. Our case series included only 7 patients because, based on these observations, subsequent patients undergoing TPVI with the Venus P-Valve were discharged on oral anticoagulation (OAC) for 3 months, followed by antiplatelet therapy, in accordance with the updated internal protocol. However, patients with pre-existing indications for anticoagulation remained on OAC indefinitely.

These findings underscore the importance of continued monitoring and further research into transcatheter pulmonary valve replacements.

## Conclusions

Our case series highlights the potential importance of antithrombotic therapy following TPVI with the Venus P-Valve. The concurrent presence of HALT and RELM raises questions about the adequacy of SAPT, which is often recommended by current guidelines. Although our findings suggest that SAPT may not be sufficient for certain high-risk patients, further investigation is necessary to explore the potential benefits of dual antiplatelet therapy or anticoagulation in this population.

Future research is warranted to: 1) validate the prevalence of HALT and RELM and identify associated risk factors in larger cohorts; 2) clarify clinical predictors of thrombotic events; 3) evaluate long-term outcomes of different antithrombotic regimens; and 4) conduct trials to better understand optimal therapy based on patient risk profiles. This research will help to deepen our understanding of HALT and its implications, refine antithrombotic strategies, and potentially improve outcomes in patients undergoing TPVI.

## Funding Support and Author Disclosures

This study was partially supported by “Ricerca Corrente” funding from Italian Ministry of Health to IRCCS Policlinico San Donato. The authors have reported that they have no relationships relevant to the contents of this paper to disclose.

## Appendix

Video 1 Computed Tomography Angiography at 6 Months of Follow-up Shows HALT in all 3 Leaflets

## References

[1] Driesen B.W., Warmerdam E.G., Sieswerda G.J. (2019). Transcatheter pulmonary valve implantation: current status and future perspectives. Curr Cardiol Rev.

[2] McInerney A., Bagur R. (2023). Hypoattenuated leaflet thickening after transcatheter aortic valve implantation: the research around HALT does not halt. Am J Cardiol.

[3] Ruile P., Jander N., Blanke P. (2017). Course of early subclinical leaflet thrombosis after transcatheter aortic valve implantation with or without oral anticoagulation. Clin Res Cardiol.

[4] Zhu W., Xia Z., Chan Shi Kai J. Feasibility of self-expanding transcatheter pulmonary valves in patients with pyramidal RVOT: favorable mid-term outcomes, 29 November 2024, PREPRINT (Version 1) available at Research Square.

[5] Saram S.J., Leong M.C., Sivalingam S. (2021). Explantation of the Venus P-valve: a first in-human experience. Interact Cardiovasc Thorac Surg.

[6] Hammadah M., Han B.K., de Oliveira Nunes M. (2021). Hypoattenuated leaflet thickening after transcatheter pulmonary valve replacement with the SAPIEN 3 valve. JACC Cardiovasc Imaging.

[7] Parekh J.D., Cheng V.Y., Baker C.M., Gössl M. (2024). Hypoattenuated leaflet thickening after transcatheter pulmonary valve replacement. JACC Cardiovasc Interv.

[8] Pilati M., Duong P., Bordonaro V., Secinaro A., Butera G. (2023). Auto-expandable pulmonary valve leaflet microthrombosis: a subtle and dangerous phenomenon. Eur Heart J Cardiovasc Imaging.

[9] Hein M., Breitbart P., Minners J. (2022). Performance of computed tomography angiography (CTA) for the diagnosis of hypo-attenuated leaflet thickening (HALT). J Clin Med.

